# A High Fat/High Sugar Diet Alters the Gastrointestinal Metabolome in a Sex Dependent Manner

**DOI:** 10.3390/metabo10100421

**Published:** 2020-10-20

**Authors:** Ayland C. Letsinger, Rani Menon, Anjushree R. Iyer, Heather L. Vellers, Jorge Z. Granados, Arul Jayaraman, J. Timothy Lightfoot

**Affiliations:** 1 The Department of Health Kinesiology, Texas A&M University, College Station, TX 77845, USA; Heather.Vellers@ttu.edu (H.L.V.); Jorge.Granados@UTSouthwestern.edu (J.Z.G.); tlightfoot@tamu.edu (J.T.L.); 2 Artie McFerrin Department of Chemical Engineering, Texas A&M University, College Station, TX 77843, USA; inarmenon@gmail.com (R.M.); anjushree.iyer@tamu.edu (A.R.I.); arulj@mail.che.tamu.edu (A.J.)

**Keywords:** western diet, fructose, cecum, metabolism, obesity, inflammation, androgen, estrogen, neurotransmitter

## Abstract

The gut metabolome offers insight for identifying the source of diet related pathology. As such, the purpose of this study was to characterize alterations of the gut metabolome in female and male C57BL/6J mice randomly assigned to a standard “chow” diet (CHOW) or a high fat/high sugar diet (HFHS; 45% fat and 20% fructose drinking solution) for nine weeks. Cecal metabolites were extracted and an untargeted analysis via LC-MS/MS was performed. Partial Least Sums Discriminate Analysis (PLS-DA) presented significant differences between the two diet groups in a sex-dependent manner. Mann–Whitney U-tests revealed 2443 and 1669 features to be significantly different between diet groups in the females and males, respectively. The majority of altered metabolites were depleted within the cecum of the HFHS fed mice. Metabolic pathways associated with galactose metabolism, leukotriene metabolism, and androgen and estrogen biosynthesis and metabolism were differentially altered with an HFHS diet between sexes. We concluded the immense metabolite depletion and elevation of adverse metabolites associated with the HFHS diet is suggestive of poor gut health. Further, the differential alterations between female and male mice suggests that sex plays an important role in determining the effect of diet on the metabolome and host health.

## 1. Introduction

Poor diet is considered the leading cause of preventable death in the United States [1] and directly contributes to low quality of life via cardiovascular disease, diabetes, obesity, and mental health disorders [2]. Recent studies have shown that nutrients can have variable effects on health due to genetic and environmental interactions. For example, single nucleotide polymorphisms within APOA5, LIPC, and CETP gene regions have been associated with altered lipid profiles in response to the same diet [3]. The interactions can also be sex dependent as females have greater incidence of irritable bowel syndrome [4] and Crohn’s disease [5], while men experience greater incidence of ulcerative colitis [5] and colon cancer [6].

Causal links between specific nutrients and health are difficult to establish due to the physiological complexity, time, and cost required for longitudinal studies. Nutritional epidemiology, while critically important for public health, provides limited information as data can be fraught with confounding variables and accuracy complications [7]. Nutrigenetic approaches have also attempted to associate nutritional outcomes with single nucleotide polymorphisms and have come up with few hits, most of which have small effects [8]. Additionally, the vast epistatic nature of the genome requires high costs and large numbers of participants to reliably detect sequences of interest. Here we present a bottom-up approach by analyzing the intestinal metabolome and identify characteristics likely to generate adverse physiological outcomes, such as inflammation, that can decrease quality of life.

Previous studies from our lab [9] have clearly shown that a diet high in fat and sugar significantly decreases daily physical activity in mice. Our previous work suggests one potential biological controller of daily physical activity is found within the limbic system via dopaminergic function [10] and in a small number of metabolic pathways [11]. Given the recent connection between gut health and brain function [12,13], we have begun to investigate the potential that alterations in gut metabolome can result in changes in behavioral markers such as daily physical activity.

Therefore, the purpose of this study was to characterize the effect of a high fat, high sugar diet on gastrointestinal nutrient digestion and metabolism between sexes that could provide evidence for altered health and behavioral outcomes. We performed an untargeted metabolite analysis via mass spectrometry coupled with liquid chromatography separation to generate a repository of altered metabolites from the cecum of C57BL/6J mice on a standard rodent diet compared to a high fat/high sugar diet (HFHS; 45% fat in hard food coupled with 20% fructose in drinking water). The compiled metabolomic data have provided a method and foundation for future studies to causally test the connection of specific metabolite production and physiological function/dysfunction as well as an understanding of sexual dichotomies in metabolism related to inflammation, steroids, sex hormones, and neurotransmitters.

## 2. Results

**Figure 1:**
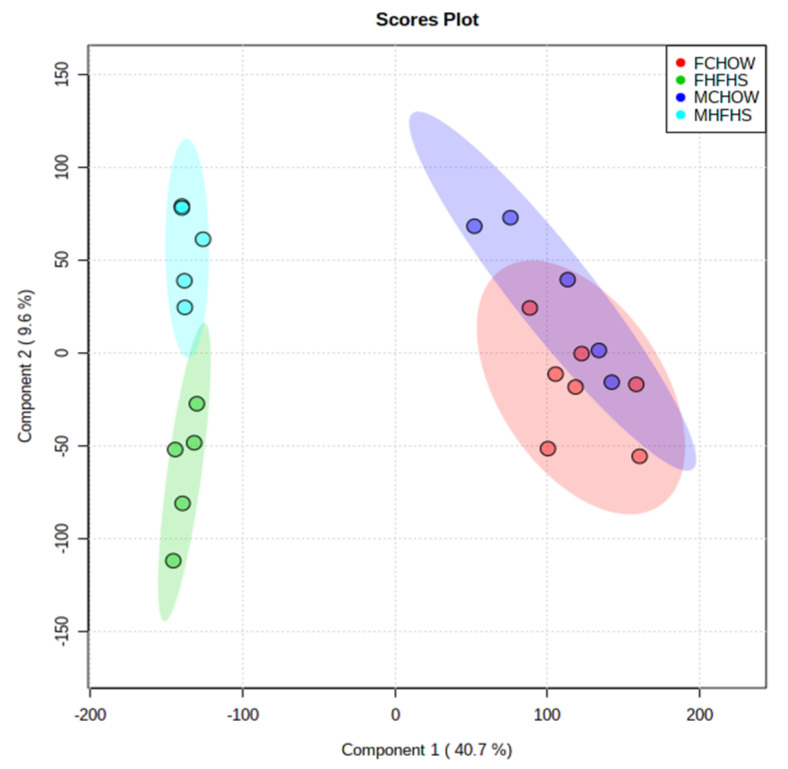
Partial Least Sums Discriminate Analysis (PLS-DA) of the Cecal Metabolome in Female and Male Mice on a CHOW or High Fat/High Sugar (HFHS) Diet. Cecal metabolites from mice on either diet were acquired using untargeted LC-MS/MS analysis and the features were clustered. FCHOW: female mice, chow diet; FHFHS: female mice high fat/high sugar diet; MCHOW: male mice, chow diet; MHFHS: male mice, high fat/high sugar diet.

**Figure 2:**
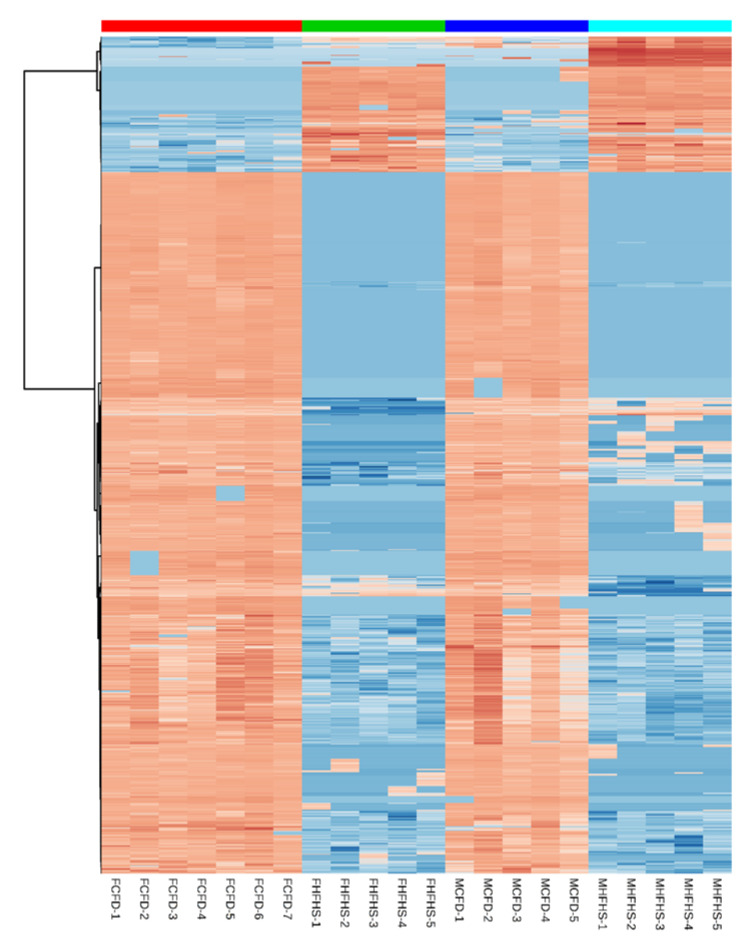
Heat Map of Top 1000 Differentially Expressed Features in Female and Male Mice on a CHOW or HFHS Diet. A total of 10,961 features were detected in cecal contents across all mice (*n* = 22). Of these, 2837 and 1669 features were significantly different between female and male mice, respectively, on either diet. A total of 147 features were significantly (*p* < 0.01) different between female and male mice on the HFHS diet. Columns represent specific mice from each group. Rows are unique features detected via LC-MS/MS. Darker red indicates relatively abundant features and dark blue indicates relatively depleted features. FCFD: female mice, chow diet; FHFHS: female mice high fat/high sugar diet; MCFD: male mice, chow diet; MHFHS: male mice, high fat/high sugar diet.

Partial Least Sums Discriminate Analysis (PLS-DA) of the untargeted cecal metabolite data presented significant differences between the two diet groups in a sex-dependent manner (Figure 1). There was little separation between female and male mice reared on the CHOW diet; however, the separation between female and male mice was more pronounced in HFHS fed mice (Figure 1). Of the 10,961 detected metabolites, the Mann–Whitney U-tests yielded 2443 and 1669 features that were significantly altered (*p* < 0.01) between diet groups in females and males, respectively. A heatmap of the top 1000 differentially altered metabolites (clustered with Ward’s method) demonstrates the unique signature of each diet and consistency within groups (Figure 2). Two clusters of metabolite expression were observed in both male and female mice on the CHOW diet: a cluster of metabolites with increased abundance and a smaller cluster of metabolites that were decreased in abundance. Conversely, the pattern of significant metabolite alteration in the HFHS fed mice was the opposite to that observed into the CHOW fed mice. The metabolite cluster that was increased in the CHOW fed mice was significantly decreased in the HFHS fed mice.

**Figure 3:**
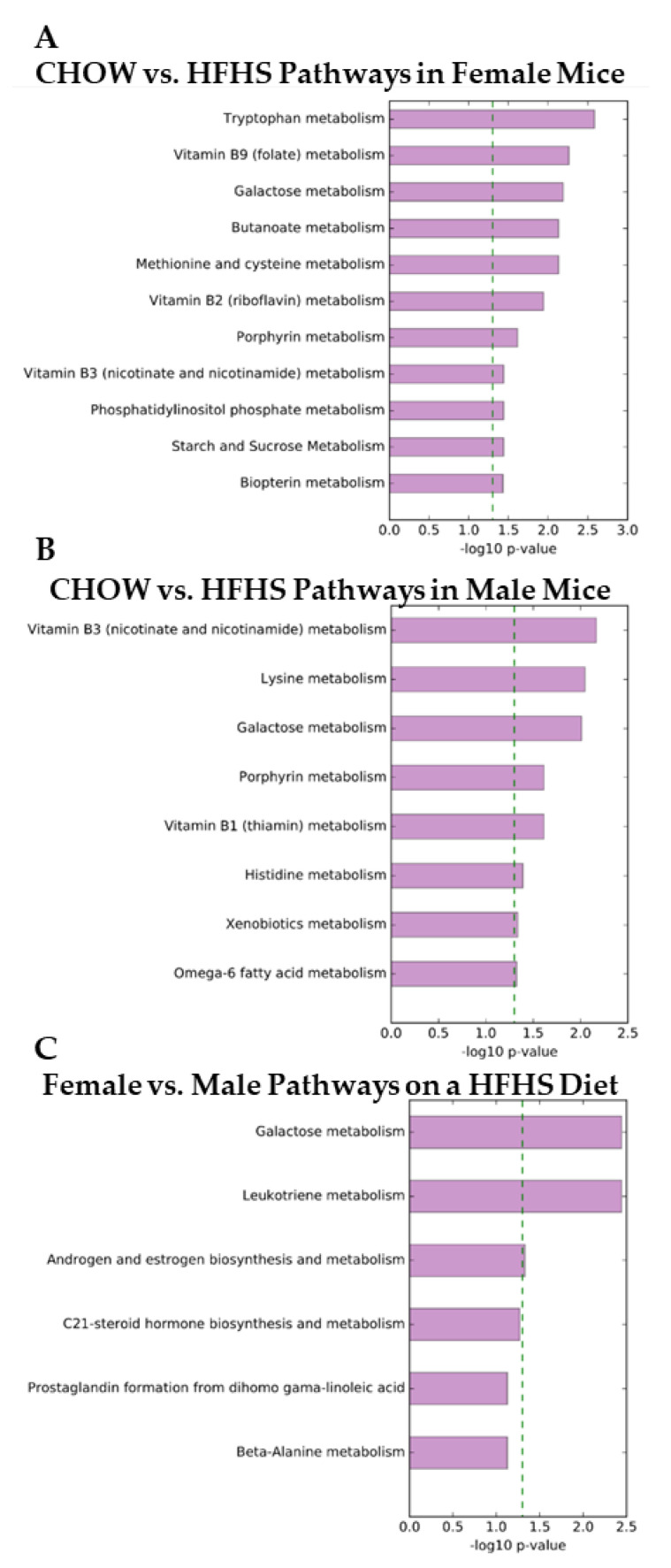
Mummichog Pathway Analysis. Generated bar graphs depict detected metabolite pathways that had altered metabolites at a significance level of *p* < 0.0025. (**A**) Eleven detected pathways were altered between diets in female mice. (**B**) Eight detected pathways were altered between diets in male mice. (**C**) Three detected pathways were altered between sexes fed an HFHS diet.

Detected metabolites within a mass accuracy of 1 ppm were compiled and analyzed within Mummichog [14], a Python program for analyzing network organization and pathway enrichment of untargeted metabolomic data based on metabolic networks from Kyoto Encyclopedia of Genes and Genomes (KEGG) [15], UCSD Recon1 [16], and the Edinburgh human metabolic network [17]. Briefly, untargeted features detected from our analysis were compared with known metabolic pathways based on reaction closeness and completeness. Once generated, feature differences of pathways between experimental group networks were statistically compared using Fisher’s exact tests with an alpha of 0.0025. Step by step methods can be found in the original publication [14]. In females, metabolites from eleven pathways were significantly altered in mice on the HFHS diet (Figure 3A). In males, metabolites from eight pathways were significantly altered by the HFHS diet (Figure 3B). Three pathways, the galactose, leukotriene, and androgen and estrogen biosynthesis metabolism, were differentially altered by the HFHS diet between sexes (Figure 3C). The only pathway found to be altered within each comparison was galactose metabolism which was expected given the differences in sugars present between the diets.

**Figure 4:**
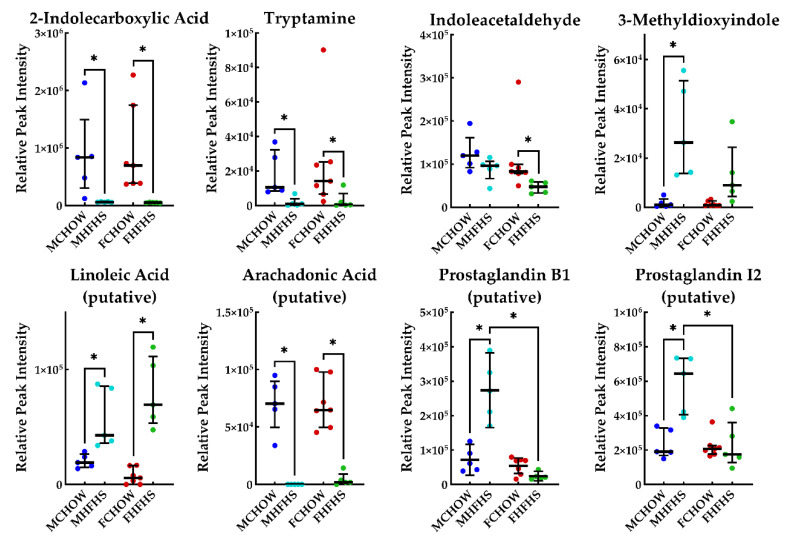
Inflammation Related Metabolites. Horizontal bars represent the median relative peak intensity of each metabolite with the interquartile range. * *p* < 0.01. MCHOW vs. FCHOW, MCHOW vs. FHFHS, and MHFHS vs. FHFHS groups were not compared.

**Figure 5:**
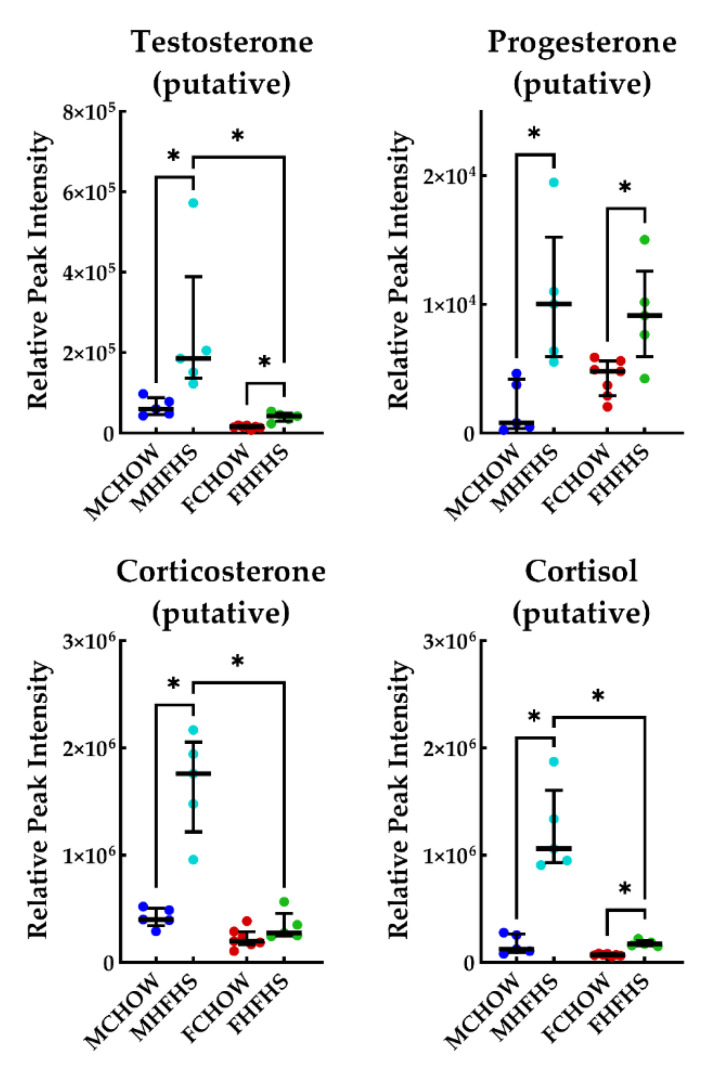
Androgen and Steroid Hormone Related Metabolites. Horizontal bars represent the median relative peak intensity of each metabolite with the interquartile range. * *p* < 0.01. MCHOW vs. FCHOW, MCHOW vs. FHFHS, and MHFHS vs. FHFHS groups were not compared.

Metabolites from significantly altered pathways of interest were then examined to better interpret specific differences between groups. Female and male mice fed the HFHS diet had depleted levels of 2-indolecarboxylic acid, tryptamine, arachadonic acid and elevated levels of 3-linoleic acid (Figure 4). Indoleacetaldehyde was depleted in female HFHS fed mice only, while 3-methyldioxyindole, prostaglandin B1, and prostaglandin I2 were elevated in male HFHS fed mice only. Testosterone, progesterone, and cortisol were elevated in both sexes fed the HFHS diet, but to a greater extent in male mice (Figure 5). Corticosterone was significantly elevated in male HFHS fed mice only. Additionally, acetylcholine was significantly elevated in CHOW fed mice (not shown; four-fold greater).

## 3. Discussion

The HFHS diet generated significant changes on the cecal metabolome of both female and male C57Bl/6J mice. Not only were there significant and clear differences in the metabolome between unique diets, but there was a sexual dimorphic response exhibited between females and males exposed to the same HFHS diet. Our data suggest that the sexual dimorphic metabolome response may partially explain why there are sex differences in gastrointestinal disorder incidence or behavioral responses [9]. Furthermore, the metabolite depletion and observed changes in metabolites associated with inflammation, steroids, sex hormones, and neurotransmitters indicates the HFHS diet likely led to an unfavorable gut environment. Therefore, not only does a diet high in fat and sugar generally alter the metabolome, there are also unique sex differences in response which may lead to differential health outcomes. As the cecal metabolome is known to be affected by the microbiome alone and in conjunction with host metabolism [18], all pathways affected in this study cannot be assumed to be a product of host metabolism alone. For example, fructose and high fat content both have independent evidence for promoting pathogenic microbiota that could explain much of the data in the following paragraphs [19,20].

### 3.1. Inflammation

A primary sign of poor intestinal health is the presence of inflammation [21]. When intestinal epithelial cells become damaged, barrier integrity is compromised, potentially leading to elevated bacterial endotoxins or harmful metabolites in the bloodstream [22]. High fat foods and fructose have both been linked to the presence of intestinal inflammation, increased epithelial permeability, and the onset of non-alcoholic fatty liver disease [23]. The addition of indole, a metabolite generated from the breakdown of tryptophan, can help mitigate this inflammation by improving barrier health [24]. From our current analysis, mice on the HFHS diet had no detectable levels of 2-indolecarboxylic acid in the cecum suggesting a lack of protection from lipid peroxidation associated inflammation in these mice (Figure 4) [21]. Additionally, CHOW fed mice had greater levels of cecal tryptamine, which recent evidence suggests can be beneficial for gut health by increasing colonic fluid and anion secretion [25]. Female CHOW fed mice had greater levels of cecal indoleacetaldehyde, although, no function is known for this metabolite (Figure 4). The only indole metabolite found in greater quantities in the HFHS fed mice was 3-methyldioxyindole, which is colloquially associated with the foul smell of feces, but not diminished inflammation or other disorders (Figure 4). Interestingly, only male mice fed an HFHS diet had elevated cecal levels of metabolites associated with tissue damage and inflammation such as prostaglandins (Figure 4) [14]. Precursors to prostaglandins such as linoleic acid (elevated in HFHS fed mice) and arachidonic acid (elevated in CHOW fed mice) do not provide adequate evidence as to why prostaglandins may be elevated in male HFHS fed mice (Figure 4). As such, this dimorphism should be investigated further.

### 3.2. Androgen Biosynthesis and Metabolism

Androgens are critical for many physiological functions affecting growth, mood, and vitality. While limited, previous studies have linked diet with alterations in serum sex hormones. Bouchard et al. overfed male subjects by 1000 kcal/day, six days a week, for 100 days resulting in depletion of serum androgens [26]. There is also evidence that endotoxemia via chronic intestinal inflammation is negatively associated with serum androgens [27]. However, increases in serum androgen levels are associated with overfeeding in females [26] indicating critical sex-dependent considerations should be made before constructing causal links. Our untargeted analysis revealed a significant increase in the production of cecal testosterone and progesterone in females and males exposed to the HFHS diet (Figure 5).

Elevated levels of cortisol were also found in the cecum of mice fed the HFHS diet (Figure 5). While cortisol is primarily metabolized in the blood, a small portion can be excreted in bile, especially in times of stress [28]. Cecal corticosterone was also found to be elevated only in male mice fed the HFHS diet. Importantly, Ridlon et al. discovered a gut microbe, *Clostridium scindens*, that can convert corticosteroids in the gut into androgens [26]. The effects of elevated androgens and steroid hormones in the gut are not well documented and, while our results cannot demonstrate a direct link with serum levels, these data provide an intriguing basis for future studies. In a previous study from our laboratory, differences in serum levels of 17β-estradiol and testosterone between diets were not detectable via ELISAs [9]. Further, corticosteroids are important modulators of intestinal inflammation which can also be reabsorbed back into circulation [29] suggesting that in HFHS fed male mice, this increase in cecal corticosterone, in addition to the metabolites discussed previously, may be another modulator of gut inflammation. 

### 3.3. Neurotransmitter Biosynthesis and Metabolism

The production of neurotransmitters in the gut has been a target of many studies attempting to connect mood states with gut function; however, the causal connection to mood states has yet to be determined [30], and generally the production of neurotransmitters is associated with gut motility and irritable bowel syndrome [31]. The HFHS diet resulted in elevated levels of both serotonin in females-only (via tryptophan metabolism) and glutamate in males-only (via lysine metabolism) associated metabolites (Figure 3A,B). The mechanisms underlying the differential expression of these pathways, while unknown, provide an intriguing basis for future studies investigating the gut–brain axis.

Further benefiting the CHOW mice, relatively high levels of acetylcholine, a metabolite of choline, was found in the cecum of mice fed a CHOW diet. Acetylcholine release via cholinergic neurons is critical for fluid shifts and secretion rate between the lumen and vasculature [32]. The presence of acetylcholine has also been shown to be anti-inflammatory and can mitigate carcinogenesis [33].

## 4. Materials and Methods

### 4.1. Experimental Timeline

The cecal contents used in this study were taken from a previously published study [9] approved by the Texas A&M University Institutional Animal Care and Use Committee (AUP 2013-0274). Briefly, at three weeks of age, C57BL/6J pups were weaned, individually housed, and randomly assigned to an intervention diet. Two ad libitum diets were utilized: a standard “chow” diet (CHOW) consisting of 4% fat, 25.2% protein, 39.5% carbohydrate, 3.3% crude fiber, 10% neutral fiber, and 9.9% ash (Harlan Labs, Houston, TX, USA) and a high fat/high sugar diet (HFHS) consisting of 45% fat, 20% protein, 35% carbohydrate, 5% fiber, and a 20% fructose solution in place of regular drinking water (product D12451, Research Diets, Inc., New Brunswick, NJ, USA; female on CHOW *n* = 7; female on HFHS *n* = 5; male on CHOW *n* = 5; male on HFHS *n* = 5). The HFHS diet was used to mimic a United States citizen with a worse than average diet as according to the National Health and Nutrition Examination Survey where the United States’ mean total fat intake is ~34% [34] and mean total calories from fructose is ~10% [35]. We have previously shown this HFHS diet significantly increases body fat in both female and male inbred C57B/6J mice, yet males intake more calories and gain more body fat [9]. This diet also significantly decreased the levels of voluntary wheel running with males’ activity levels decreasing ≈70%, while females’ activity decreased ≈57% across a 9–11 week period [9]. During the initial study, caloric intake was measured weekly using an electronic scale. Body composition was measured weekly using magnetic resonance imaging (EchoMRI, Houston, TX, USA). During sacrifice of these animals, cecal contents were squeezed into a cryotube, immediately flash frozen in liquid nitrogen, and stored long-term at −80 °C.

Due to the increased caloric intake of the ad libitum HFHS compared to an ad libitum CHOW diet, we termed the HFHS diet intervention “overfeeding”. Inbred C57BL/6J were selected as the best model for human translation due to their susceptibility to diet induced obesity [36], slight preference for fructose water over regular water [37], and their consistent and repeatable wheel running phenotype [38,39].

### 4.2. Metabolite Extraction

Metabolites in samples from Vellers et al. [9] were extracted using a solvent-based method [18]. Ice-cold methanol/chloroform (2:1, *v*/*v*) was added to pre-weighed cecal samples. Samples were homogenized in a Precellys 24 homogenizer using garnet bead tubes at 5000 g for 20 s. After homogenization, the samples were centrifuged at 3000× *g* for 10 min at 4 °C and the supernatant was collected. A second sequential extraction was performed on the pellet to maximize extraction of metabolites and the supernatants from the two extractions were pooled. Ice-cold water (600 µL) was then added to the supernatants, vortexed and centrifuged at 5000× *g* for 5 min at 4 °C to obtain phase separation. Both the upper and lower phases were collected. The upper phase was passed through a 0.2 micron filter and then lyophilized. The concentrated samples were resuspended in 200 µL of methanol/water (1:1 *v*/*v*) and stored at −80 °C.

Untargeted metabolomic analysis was carried out using a hybrid quadrupole-Orbitrap mass spectrometer (Q Exactive, Thermo Scientific, Waltham, MA) coupled to a UHPLC system (Dionex UltiMate 3000, Thermo Scienrific). A C18 Synergi Fusion-RP 4 µ 80Å 150 × 2.0 mm column (Phenomenex) was used for chromatographic separation with 0.1% formic acid in water (Solvent A) and with 0.1% formic acid in ethanol (Solvent B). MS1 and MS1-dependent MS2 spectra was collected at a m/z resolution of 37,500. Metabolites were eluted at a flow rate of 0.4 mL/min. The flow gradient was 40% of solvent B for 5 min, 95% of solvent B for 7 min and 10% solvent B for 8 min. Blanks (methanol and water at 1:1 v/v) were inserted between every sample to prevent any sample carryover. Deuterated indole-3-acetic acid was used as a labeled internal quality control standard. Pure standards of metabolites of interest were used to generate a standard curve for absolute quantification. Data were analyzed using Progenesis QI software (Waters), the Human Metabolome Database (HMDB), and the Kyoto Encyclopedia of Genes and Genomes (KEGG) databases for metabolite identification. Raw abundance data were normalized to fecal sample weights and statistical analysis was performed using KaleidaGraph (Synergy). Pathway analysis was done using Mummichog with default parameters [14].

### 4.3. Statistical Analysis

After untargeted detection of positive, monoisotopic ions peaks via mass spectrometry, median metabolite levels between groups of interest (male CHOW vs. male HFHS, female CHOW vs. female HFHS, and male HFHS vs. female HFHS) were analyzed with the non-parametric Mann–Whitney U-test with an alpha level of 0.01.

## 5. Conclusions

The findings of our untargeted analysis have characterized distinct changes in the cecal metabolome of mice fed an HFHS diet. It is clear, at least from the murine perspective, that an HFHS diet promotes poor gut health via alterations in metabolites associated with damage and inflammation, androgens, and neurotransmitters. These findings are important as depleted metabolites critical for host health could potentially be identified and replenished by supplementation while simultaneously removing nutrients that lead to formation of pro-inflammatory molecules. Additionally, the variance in metabolomic signatures between sexes should be considered while investigating the effects of diet on the metabolome and host health in future studies.

## References

[1] Mokdad A.H., Ballestros K., Echko M., Glenn S., Olsen H.E., Mullany E., Lee A., Khan A.R., Ahmadi A., Ferrari A.J. (2018). The State of US Health, 1990–2016. JAMA.

[2] Wang D.D., Li Y., Afshin A., Springmann M., Mozaffarian D., Stampfer M.J., Hu F.B., Murray C.J.L., Willett W.C. (2019). Global Improvement in Dietary Quality Could Lead to Substantial Reduction in Premature Death. J. Nutr..

[3] Heianza Y., Qi L. (2017). Gene-Diet Interaction and Precision Nutrition in Obesity. Int. J. Mol. Sci..

[4] Saito Y.A., Schoenfeld P., Locke G.R. (2002). The Epidemiology of Irritable Bowel Syndrome in North America: A Systematic Review. Am. J. Gastroenterol..

[5] Loftus E.V. (2004). Clinical Epidemiology of Inflammatory Bowel Disease: Incidence, Prevalence, and Environmental Influences. Gastroenterology.

[6] Haggar F., Boushey R. (2009). Colorectal Cancer Epidemiology: Incidence, Mortality, Survival, and Risk Factors. Clin. Colon Rectal Surg..

[7] Lilienfeld A.M. (1983). Practical Limitations of Epidemiologic Methods. Environ. Health Perspect..

[8] Loos R.J.F. (2019). From Nutrigenomics to Personalizing Diets: Are We Ready for Precision Medicine?. Am. J. Clin. Nutr..

[9] Vellers H.L., Letsinger A.C., Walker N.R., Granados J.Z., Lightfoot J.T. (2017). High Fat High Sugar Diet Reduces Voluntary Wheel Running in Mice Independent of Sex Hormone Involvement. Front. Physiol..

[10] Knab A.M., Bowen R.S., Hamilton A.T., Gulledge A.A., Lightfoot J.T. (2009). Altered Dopaminergic Profiles: Implications for the Regulation of Voluntary Physical Activity. Behav. Brain Res..

[11] Ferguson D.P., Dangott L.J., Vellers H.L., Schmitt E.E., Lightfoot J.T. (2015). Differential Protein Expression in the Nucleus Accumbens of High and Low Active Mice. Behav. Brain Res..

[12] Arneth B.M. (2018). Gut-Brain Axis Biochemical Signalling from the Gastrointestinal Tract to the Central Nervous System: Gut Dysbiosis and Altered Brain Function. Postgrad. Med. J..

[13] Strandwitz P. (2018). Neurotransmitter Modulation by the Gut Microbiota. Brain Res..

[14] Li S., Park Y., Duraisingham S., Strobel F.H., Khan N., Soltow Q.A., Jones D.P., Pulendran B. (2013). Predicting Network Activity from High Throughput Metabolomics. PLoS Comput. Biol..

[15] Kanehisa M., Goto S., Hattori M., Aoki-Kinoshita K.F., Itoh M., Kawashima S., Katayama T., Araki M., Hirakawa M. (2006). From Genomics to Chemical Genomics: New Developments in KEGG. Nucleic Acids Res..

[16] Duarte N.C., Becker S.A., Jamshidi N., Thiele I., Mo M.L., Vo T.D., Srivas R., Palsson B. (2007). Global Reconstruction of the Human Metabolic Network Based on Genomic and Bibliomic Data. Proc. Natl. Acad. Sci. USA.

[17] Ma H., Sorokin A., Mazein A., Selkov A., Selkov E., Demin O., Goryanin I. (2007). The Edinburgh Human Metabolic Network Reconstruction and Its Functional Analysis. Mol. Syst. Biol..

[18] Sridharan G.V., Choi K., Klemashevich C., Wu C., Prabakaran D., Pan L.B., Steinmeyer S., Mueller C., Yousofshahi M., Alaniz R.C. (2014). Prediction and Quantification of Bioactive Microbiota Metabolites in the Mouse Gut. Nat. Commun..

[19] Volynets V., Louis S., Pretz D., Lang L., Ostaff M.J., Wehkamp J., Bischoff S.C. (2017). Intestinal Barrier Function and the Gut Microbiome Are Differentially Affected in Mice Fed a Western-Style Diet or Drinking Water Supplemented with Fructose. J. Nutr..

[20] Sellmann C., Priebs J., Landmann M., Degen C., Engstler A.J., Jin C.J., Gärttner S., Spruss A., Huber O., Bergheim I. (2015). Diets Rich in Fructose, Fat or Fructose and Fat Alter Intestinal Barrier Function and Lead to the Development of Nonalcoholic Fatty Liver Disease over Time. J. Nutr. Biochem..

[21] Derikx J.P. (2010). Non-Invasive Markers of Gut Wall Integrity in Health and Disease. World J. Gastroenterol..

[22] Schippa S., Conte M. (2014). Dysbiotic Events in Gut Microbiota: Impact on Human Health. Nutrients.

[23] Frazier T.H., DiBaise J.K., McClain C.J. (2011). Gut Microbiota, Intestinal Permeability, Obesity-Induced Inflammation, and Liver Injury. J. Parenter. Enter. Nutr..

[24] Whitfield-Cargile C.M., Cohen N.D., Chapkin R.S., Weeks B.R., Davidson L.A., Goldsby J.S., Hunt C.L., Steinmeyer S.H., Menon R., Suchodolski J.S. (2016). The Microbiota-Derived Metabolite Indole Decreases Mucosal Inflammation and Injury in a Murine Model of NSAID Enteropathy. Gut Microbes.

[25] Xiao C., Cho J.R., Zhou C., Treweek J.B., Chan K., McKinney S.L., Yang B., Gradinaru V. (2016). Cholinergic Mesopontine Signals Govern Locomotion and Reward through Dissociable Midbrain Pathways. Neuron.

[26] Kokkinos P., Myers J., Faselis C., Panagiotakos D.B., Doumas M., Pittaras A., Manolis A., Kokkinos J.P., Karasik P., Greenberg M. (2010). Exercise Capacity and Mortality in Older Men. Circulation.

[27] Tremellen K., McPhee N., Pearce K., Benson S., Schedlowski M., Engler H. (2018). Endotoxin-Initiated Inflammation Reduces Testosterone Production in Men of Reproductive Age. Am. J. Physiol. Metab..

[28] Palme R., Rettenbacher S., Touma C., El-Bahr S.M., Mostle E. (2005). Stress Hormones in Mammals and Birds: Comparative Aspects Regarding Metabolism, Excretion, and Noninvasive Measurement in Fecal Samples. Ann. N. Y. Acad. Sci..

[29] Morris D.J., Brem A.S. (2019). Role of Gut Metabolism of Adrenal Corticosteroids and Hypertension: Clues Gut-Cleansing Antibiotics Give Us. Physiol. Genom..

[30] Johnson K.V., Foster K.R. (2018). Why Does the Microbiome Affect Behaviour?. Nat. Rev. Microbiol..

[31] Mittal R., Debs L.H., Patel A.P., Nguyen D., Patel K., O’Connor G., Grati M., Mittal J., Yan D., Eshraghi A.A. (2017). Neurotransmitters: The Critical Modulators Regulating Gut-Brain Axis. J. Cell. Physiol..

[32] Keely S.J. (2011). Epithelial Acetylcholine—A New Paradigm for Cholinergic Regulation of Intestinal Fluid and Electrolyte Transport. J. Physiol..

[33] Tracey K.J. (2007). Physiology and Immunology of the Cholinergic Antiinflammatory Pathway. J. Clin. Investig..

[34] Ford E.S., Dietz W.H. (2015). Trends in Energy Intake among Adults in the United States: Findings from NHANES. HHS Public Access.

[35] Vos M.B., Kimmons J.E., Gillespie C., Welsh J., Blanck H.M. (2008). Dietary Fructose Consumption among US Children and Adults: The Third National Health and Nutrition Examination Survey. Medscape J. Med..

[36] Collins S., Martin T.L., Surwit R.S., Robidoux J. (2004). Genetic Vulnerability to Diet-Induced Obesity in the C57BL/6J Mouse: Physiological and Molecular Characteristics. Physiol. Behav..

[37] Glendinning J.I., Breinager L., Kyrillou E., Lacuna K., Rocha R., Sclafani A. (2010). Differential Effects of Sucrose and Fructose on Dietary Obesity in Four Mouse Strains. Physiol. Behav..

[38] Knab A.M., Bowen R.S., Moore-harrison T., Hamilton A.T., Turner J., Lightfoot J.T. (2010). Repeatability of Exercise Behaviors in Mice. Physiol. Behav..

[39] Lightfoot J.T., Turner M.J., Pomp D., Kleeberger S.R., Leamy L.J. (2008). Quantitative Trait Loci for Physical Activity Traits in Mice. Physiol. Genom..

